# Reply to Schäfers

**DOI:** 10.1093/icvts/ivad060

**Published:** 2023-05-05

**Authors:** Henrik Bjursten, Daniel Oudin Åström, Igor Zindovic

**Affiliations:** Lund University, Department of Cardiothoracic Surgery, Skåne University Hospital, Lund, Sweden; Division of Occupational and Environmental Medicine, Department of Laboratory Medicine, Lund University, Lund, Sweden; Division of Sustainable Health, Department of Public Health and Clinical Medicine, Umeå University, Umeå, Sweden; Lund University, Department of Cardiothoracic Surgery, Skåne University Hospital, Lund, Sweden

**Keywords:** Aortic dissection, Lunar phases

Dear Colleagues,

We thank the authors for their thoughts on our article ‘Once after a full moon: acute type A aortic dissection (ATAAD) and lunar phases’ [1]. The relation between varying acute illnesses and calendrical, geographical and meteorological phenomena has been investigated extensively lately. In addition to the current article, we have used the NORCAAD registry to investigate the relation between ATAAD and national holidays/weekends and temperature [2, 3]. We observed that weekends and national holidays had a lower incidence of surgery for ATAAD and we found that cold (<−5°C) and warm (>21°C) temperatures seem to be associated with an increase in surgery for ATAAD. Although theoretically intriguing, we wish to be humble and transparent regarding the limited clinical implication of our findings.

Hypertension is the strongest risk factor for ATAAD [4] and we believe that increased blood pressure is the common denominator for the findings in our studies. The authors suggest that large seasonal variation in daylight in the Nordic countries may be a potential confounder. However, due to the case-crossover design of the current study, individual time-invariant confounders are adjusted for by design. Control days were selected within the same month and year as the date of surgery and were matched on the day of the week. Therefore, daylight variations are unlikely to have had an effect on our results.

We agree that the finding of an increased incidence of ATAAD after full moon could be both fortuitous and unexplainable and therefore sought to find an explanation beyond the lunar phases *per se*. For instance, Cajochen *et al.* [5] demonstrated that during full moon, deep sleep was reduced by 30%, time to falling asleep was 5 min longer and total sleep time was reduced by 20 min. In turn, Lusardi *et al.* [6] showed that sleep deprivation leads to a 5- to 15-mmHg increase in systolic blood pressure and a 35% increase in urine levels of norepinephrine in hypertensive patients.

Finally, the nature and strength of our findings limits the clinical relevance of the study, and the results should not be regarded as definite but as an inspiration to think beyond the obvious pathophysiological mechanisms of disease. We now realize that our curiosity is shared by others.
